# Computed Tomography and Fluorescence Spectroscopy Blood Plasma Analysis Study for Kynurenic Acid as a Diagnostic Approach to Chronic Coenurosis in Sheep

**DOI:** 10.3390/life14091121

**Published:** 2024-09-05

**Authors:** Loredana Elena Olar, Vasile Daniel Tomoiagă, Sorin Marian Mârza, Ionel Papuc, Ioan Florin Beteg, Petru Cosmin Peștean, Mihai Musteață, Caroline Maria Lăcătuș, Raluca Marica, Paula Maria Pașca, Robert Cristian Purdoiu, Radu Lăcătuș

**Affiliations:** 1 Faculty of Veterinary Medicine, University of Agricultural Sciences and Veterinary Medicine, 400372 Cluj-Napoca, Romania; loredana.olar@usamvcluj.ro (L.E.O.); cosmin.pestean@usamvcluj.ro (P.C.P.); raluca.marica@usamvcluj.ro (R.M.);; 2 Life Sciences Institute, 3-5 Manastur Avenue, 400372 Cluj-Napoca, Romania; 3 Clinics Department, Faculty of Veterinary Medicine, University of Agricultural Sciences and Veterinary Medicine, 700490 Iasi, Romania

**Keywords:** coenurosis, plasma, kynurenic acid, fluorescence spectroscopy

## Abstract

Coenurosis is a parasitic disease caused by the larval stage of *Taenia multiceps*, *Coenurus cerebralis*, typically found in the central nervous system of different livestock such as sheep and goats. The blood plasma from fifteen clinically healthy sheep and six sheep with neurological symptoms was studied by fluorescence spectroscopy in order to establish the contribution of kynurenic acid (KYNA), the neuroprotective metabolite of the kynurenine pathway, to the total fluorescence of the plasma. CT scans were used to confirm the presence of cysts in the central nervous system of sheep with neurological symptoms. The fluorescence spectroscopy analysis and further spectra deconvolution process revealed some significantly lower KYNA contributions to the total plasma fluorescence in sheep with coenurosis compared to healthy controls. Our results indicate that KYNA emission parameters could serve as valuable diagnostic markers, particularly for detecting preclinical cases of coenurosis, thus allowing for improved farm management practices.

## 1. Introduction

**Figure 1:**
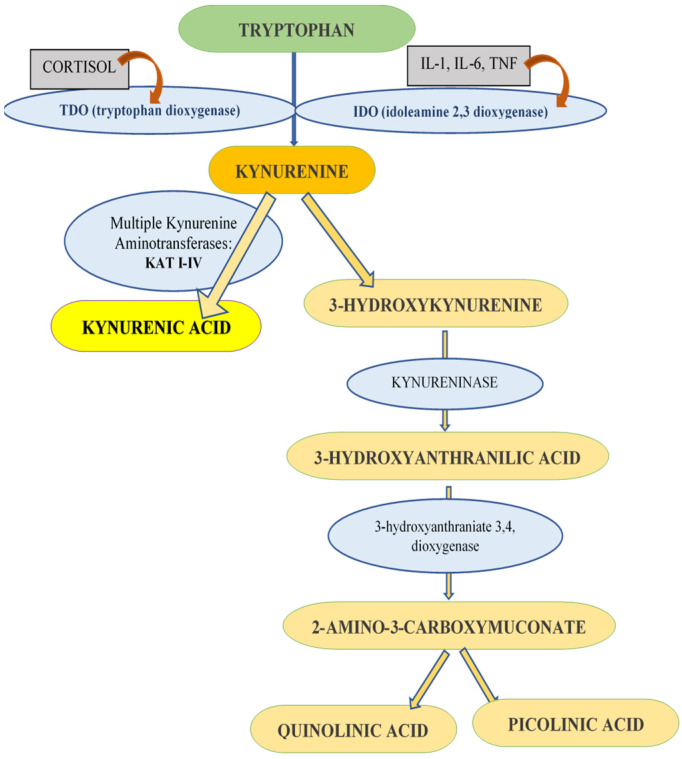
The catabolism of tryptophan via the kynurenine pathway. Yellow represents one of the most studied metabolites of kynurenine, kynurenic acid, which is assayed in the presented study. The enzymes responsible for the formation of kynurenic acid from kynurenine are called kynurenine aminotransferase (KAT I-IV).

Coenurosis is one of the most widespread neuropathological diseases in sheep, cattle, and goats, caused by *Coenurus cerebralis*, the larval stage of *Taenia multiceps* that inhabits the small intestine of definitive host such as dogs, wolves, and foxes. Transmission may occur via non-dewormed dogs that spread the adult tapeworm eggs on pasture [1]. According to some recent studies, the prevalence of coenurosis worldwide varies from 1.3% to 28.2%; a higher prevalence was reported in Italy, whereas in other European countries, such as Ireland, the UK, France and Greece, the rate of infections is lower [1,2]. In Romania, the prevalence of this disease is not yet established; studies are still needed in this area. Currently in Romania, there are still cases of sheep being diagnosed with coenurosis using the anamnesis and clinical manifestations, and confirmed by the post-mortem findings. Two clinical forms of coenurosis were reported in sheep—the acute and chronic conditions. Broadly speaking, the chronic form was more often described compared to the acute form [2]. Clinical signs of acute coenurosis depend on the number of migrating oncospheres in the CNS and are related to a toxic and allergic reaction rather than to a mechanical action of the oncospheres [1]. Furthermore, the clinical signs in chronic form are the consequence of the development of *Coenurus* in the cerebrum, cerebellum, or spinal cord [3,4]. Therefore, a variety of neurological symptoms were described in sheep and goats such as ataxia, incoordination, drowsiness, paralysis, muscle weakness, head compression, rotation, blurred vision, blindness and lack of a direct light reflex in the pupil, bruxism, poor appetite, seizures, and coma [1,2]. The best diagnostic method for cerebral coenurosis involves the interpretation of clinical signs with accurate localization of the cyst using diagnostic imaging techniques [1,5]. However, imaging techniques play an important role in confirming the diagnosis of coenurosis but remain expensive; thus, the need for reliable and less expansive tools is mandatory. Currently, spectroscopy techniques are being used in many medical subspecialties as an essential diagnostic tool that can help in designing and interpreting clinical and therapeutic trials [6,7,8,9]. Fluorescence spectroscopy is a widespread phenomenon because of the autofluorescence properties of various biological fluids and tissues of the body [6]. Body fluids and tissue fluorophores are produced in excess or decreased as a result of a certain pathology [6]. One of the fluorophores whose contribution in biological fluids can suffer some changes as a result of the presence of some neurological disorders is the neuroprotective metabolite of the kynurenine pathway (KP) produced by astrocytes and neurons, kynurenic acid (KYNA) [10,11,12]. The degradation of tryptophan is possible via the KP, which can be regulated by stress and immunocytokines (Figure 1) [13]. The KP is responsible for the neosynthesis of KYNA and picolinic, and quinolinic acids (QUIN) [11]. The two main metabolites of KP, KYNA, and QUIN are recognized as having an antagonistic effect [14]. Therefore, while KYNA is known as a neuroprotective and anticonvulsant metabolite, QUIN is recognized for its pathological effects, in particular for its ability to model neuropathological, neurochemical, and clinical features of human neurological diseases (i.e., Huntington’s disease and temporal lobe epilepsy) [14,15,16].

The study aims to investigate the potential of KYNA as a diagnostic marker for chronic coenurosis in sheep, with emphasis on its practical application in early-stage diagnosis at the farm level. Firstly, we evaluated the clinical manifestation of the sheep with coenurosis and performed computed tomography (CT) in order to determine the location of the cysts. Secondly, in order to better understand the KYNA pathway neurobiology in health and coenurosis, we evaluated the blood plasma samples through fluorescence spectroscopy to investigate both the qualitative and quantitative contribution of KYNA to the total plasma samples in clinically healthy sheep and in sheep with chronic coenurosis confirmed by CT.

## 2. Materials and Methods

### 2.1. Animals

This study was conducted on a flock of 21 sheep belonging to a meat sheep flock reared in a semi-intensive production system. Six sheep aged between 6 months and 2 years old (two males and four females) with symptoms of head pressing, unstable gait, muscle weakness, loss of herd instinct, depression lethargy and bilateral reduction in the menace reaction were referred to the Veterinary Hospital of University of Agricultural Sciences and Veterinary Medicine of Cluj-Napoca from January 2022 to January 2024. Fifteen clinically healthy sheep (ten females and five males) from the same group were used as a control group, and their ages ranged from 6 months to 3 years. In clinical examinations, vital signs were in normal ranges, while neurological response and reflexes were decreased in the affected cases. The groups were clearly defined as “A—without neurological symptoms” and “B—with neurological symptoms” to facilitate comparison in the study. The owner stated that the flock was vaccinated and dewormed annually in accordance with the comprehensive general action plan established by the National Sanitary Veterinary and Food Safety Authority. No treatment was applied to the animals before their arrival at the hospital. Blood samples were taken from the jugular veins of the sheep and transferred into EDTA/K2-containing tubes to further perform the spectrofluorimetric analysis.

### 2.2. The Fluorescence of Blood Plasma Samples

The spectrofluorometric analysis of the blood plasma samples was conducted at the spectroscopy laboratory of the Life Sciences Institute “King Michael I of Romania” in Cluj-Napoca. The fluorescence spectra of the plasma samples were recorded by using a fluorescence spectrophotometer (Jasco FP-8200 ABL&E-JASCO, Budapest, Hungary). A quantity of 1 mL of blood plasma was analyzed in the 1 cm cuvette of the instrument. The fluorescence of KYNA was measured with an excitation of 343 nm and emission of 388 nm [10]. The obtained spectra were exported in OriginPro Version 8.5.1 Software (Origin, OriginLab, Northampton, MA, USA) for peak reading and further for the deconvolution process of the bands [8,17,18]. Therefore, with the peak analyzer option of the Origin Software, we fitted the peaks and identified the band associated with the presence of KYNA in the blood plasma. After each deconvolution, a report was generated with detailed information about the fitting process and each peak, including the peak at ~388 nm associated with the emission of KYNA.

### 2.3. Computed Tomography (CT)

CT examination of the head was performed in sternal recumbency, using a multidetector 16-slice CT scanner (Siemens Somatom Scope, Munich, Germany) on helical scan mode with the following parameters: 130 kVp, 200 mAs, 1.25 slice thickness. Pre- and post-contrast delayed-phase examination was performed using a power injector (Mallinckrodt LF Dual Head CT Injector Optistat with OptiBolus, Dublin, Ireland) to infuse a bolus of 1.5 mL/kg of iodinated non-ionic contrast medium (Iohexol, Omnipaque, 350 mg L/mL, GE Healthcare, Oslo, Norvegia). Images were acquired in a soft tissue reconstruction algorithm 45 s after the contrast medium. CT of the sheep’s head was taken in axial view, and a multiplanar reconstruction was obtained in the image post-processing stage.

### 2.4. Statistical Analysis

A statistical analysis was conducted using IBM SPSS 29 Statistics. We conducted descriptive statistics to determine the mean and standard deviation for each parameter associated with the ~388 nm band, corresponding to the emission of KYNA.

An independent samples *t*-test was used to compare the parameters of the KYNA emission peak between control sheep and sheep with coenurosis. All results were considered statistically significant at a *p* value ˂ 0.05.

## 3. Results

### 3.1. Fluorescence Spectroscopy Plasma Analysis

**Figure 2:**
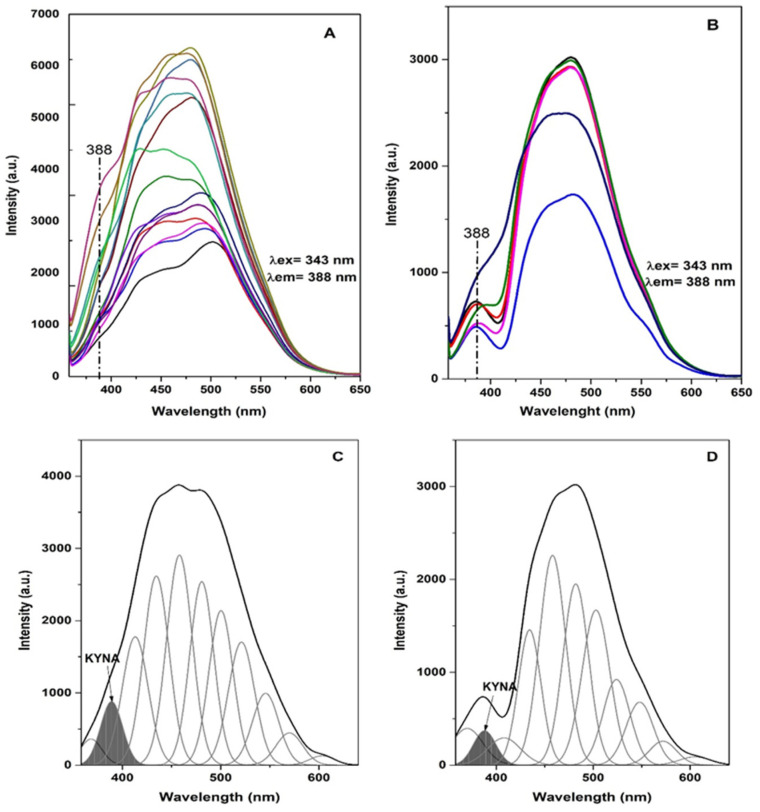
The fluorescence spectra of the control (**A**) and experimental groups (**B**) of sheep blood plasma samples. Deconvolution of the plasma fluorescence spectra belonging to a control sheep (**C**) and, respectively, to a sheep with neurological symptoms associated with coenurosis (**D**).

The fluorescence spectra of blood plasma samples collected from control sheep and, respectively, sheep with neurological symptoms associated with coenurosis are presented in Figure 2A,B. Therefore, the area of the band at 388 nm, associated with the emission of KYNA, in the spectra of the plasma samples from the sheep with clinical signs of coenurosis is modified compared with the corresponding profile of the control. Further, after the process of decomposing the peaks, we obtained the relative contribution of KYNA to the total fluorescence of plasma in clinically healthy sheep and sheep with coenurosis (Figure 2C,D). The results of the process of decomposing peaks in the spectra of the control and experimental groups of sheep concerning the band at ~388 nm are presented in Table 1. 

Therefore, the results show that the contribution of the KYNA to the total fluorescence of the blood plasma is significantly higher in the control group compared to the experimental group of sheep, as the descriptive statistics confirmed (Table 2).

The *t*-test revealed a statistically significant difference in the integrated area of the emission peak (AREA INT) between control sheep (A) and sheep with coenurosis (B) (*t* = 2.838, *p* = 0.011). The integrated area was significantly higher in control sheep, indicating elevated KYNA levels in this group.

There was no statistically significant difference in the full width at the half maximum (FWHM) of the emission peak between the two groups (*t* = 1.257, *p* = 0.224). This suggests that the width of the KYNA emission peak remains consistent regardless of the presence of coenurosis.

The maximum height of the emission peak (MAX HEIGHT) was significantly higher in control sheep compared to those with coenurosis (*t* = 3.069, *p* = 0.006). This further supports the observation that KYNA levels are elevated in healthy sheep.

The proportion of the integrated area (AREA INT P) of the emission peak relative to the total fluorescence was significantly higher in control sheep (*t* = 2.729, *p* = 0.013). This indicates that the relative contribution of KYNA to the total blood plasma fluorescence is reduced in sheep with coenurosis.

### 3.2. CT

**Figure 3:**
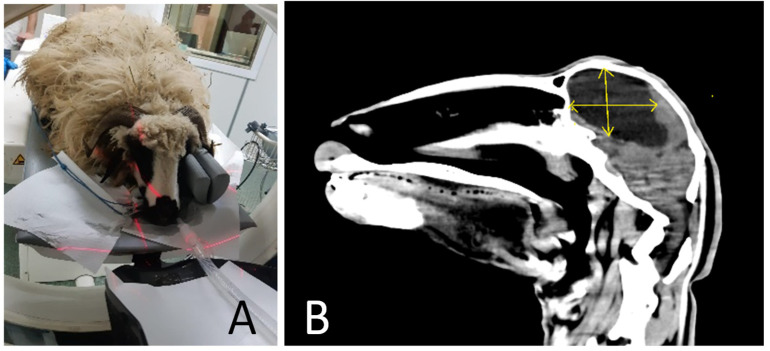
A CT scan of sheep (**A**). CT of a sheep brain affected by chronic coenurosis, cyst marked with yellow arrows (**B**).

CT confirmed the presence of cysts that are mostly extra axial, supratentorial, and produce a severe mass effect on the adjacent brain parenchyma and cerebral ventricular system (Figure 3A,B). The cysts are partially well defined, and the border definition is lost when overlaying or compressing the lateral ventricles. The content is homogeneous and izoattenuated compared to the CSF (Figure 3B).

## 4. Discussion

Currently, to the authors’ knowledge, there are no studies about the spectrofluorimetric analysis of kynurenine (KYN) neuroprotective metabolites such as KYNA in blood plasma samples collected from sheep with evidence of neurological dysfunctions associated with the presence of a coenurus cyst in a ventricle or cerebral aqueduct. The diagnosis of chronic coenurosis currently is centered on relatively new imaging techniques such as CT and MRI, which were also proposed as the gold standard methods for this condition [4,19]. Our results are in agreement with previous research; CT remains a valuable non-invasive technique to diagnose encephalic conditions of sheep. Alternatively, when planning to use this modern diagnostic imaging technique in coenurosis, a clinician should also take into account the limited finances of many sheep breeders and further recommend other techniques before this one, such as fluorescence spectroscopy, which is shown to be an economical and sensitive diagnostic tool with high efficiency and sensitivity compared to many routine medical diagnostic tools for many disorders [6].

The findings from the fluorescence spectroscopy analysis indicate significant differences in several parameters of the KYNA emission peak between control sheep and those with coenurosis. Specifically, the integrated area, maximum height and proportional area of the KYNA emission peak were significantly higher in control sheep. These results suggest that KYNA levels are elevated in healthy sheep and reduced in those affected by coenurosis. The consistent peak width (FWHM) between the groups indicates that the shape of the emission peak does not vary significantly with the condition.

In recent years, several literature reports have become available on the quantitative and qualitative research of KYN metabolites in different neurological disorders in animals and humans [13,14]. The two neuroactive KP metabolites, QUIN and KYNA, were the most investigated. Therefore, studies have shown that a reduction in KYNA levels or an abnormal increase in the QUIN–KYNA ratio in the brain can lead to neuronal damage and further determine the emergence of neurological clinical manifestations [14]. However, as other authors declared, concerns exist regarding the KP metabolites, especially for KYNA and QUIN, due to the lower concentrations of those metabolites in the brain compared to those needed to model neurological diseases acutely in rodents or primates in vivo [14]. The results of the present study show that in sheep with central nervous system lesions associated with chronic coenurosis, there is a reduction in KYNA plasma levels when compared to control animals. This difference might be a consequence of the chronic neuroinflammation from coenurosis, which can activate tryptophan depletion and further enhance cerebral KP to the detriment of the serotonin pathway known as a regulator of the nervous system [13,20,21]. Hypothetically, increased KYN synthesis from tryptophan can be enhanced by mechanisms underlying neurological inflammation such as brain infiltration with circulating immune cells, activation of resident microglia and other non-neuronal cells and brain influx of blood-derived, pro-inflammatory cytokines and other immune activators [14,22,23]. Overall, therefore, our findings concerning the lower plasma contribution of KYNA to the total fluorescence of blood plasma in sheep with coenurosis compared to control sheep might suggest that once the KP is activated by the above-mentioned mechanisms of neuro-inflammation, the expression of kynurenine aminotransferase (KAT I-IV) enzymes critical for KYNA production is reduce. Decreased plasma levels of KYNA and a diminished expression of KAT have also been described in people with type 2 diabetes, comorbid to severe mental illness [24,25]. Furthermore, a disturbance within the KP with decreased levels of KYNA and increased levels of QUIN was also associated with the aggravation of a local inflammatory response and carotid artery atherosclerosis among patients with cardiovascular disease [13,26].

Therefore, the brain surplus of KYNA negatively impacts these neurotransmitter systems and further contributes to cognitive dysfunction, as signaled in a significant number of neurodegenerative diseases and psychotic disorders in humans [14,27 28]. Interestingly, in patients with schizophrenia, while brain KYNA levels are increased, the plasma levels of KYNA are decreased, highlighting the existence of an overall dissociation between the blood and central KP metabolites [24,27,28]. In our study, the peripheral findings concerning KYNA contributions in coenurosis cannot be extrapolated to the KYNA contributions in the brain or cerebrospinal fluid; however, this hypothesis needs to be tested in future studies. Hence, our findings suggest that KYNA emission parameters could be used as reliable diagnostic markers for chronic coenurosis in sheep. While the diagnostic tool detects animals with clinical symptoms, its application could extend to identifying preclinical cases at the farm level, which could lead to earlier intervention and improved management. By detecting infections before severe clinical signs develop, farmers could implement targeted deworming strategies for definitive hosts (dogs), thus reducing the spread of the disease within flocks. Future studies will explore the mechanistic pathways of KYNA in coenurosis and validate its use as a biomarker under controlled experimental conditions. 

Several aspects of our study design may have influenced the results and conclusions. Notably, the study was conducted on sheep naturally infected with coenurosis, with animals at different stages of the disease and varying intensities of infection, which could have affected the test outcomes. To mitigate the impact of these variables, future studies will focus on experimentally infected sheep, allowing for a more controlled examination of KYNA’s contribution to the total fluorescence of plasma across different stages of coenurosis. This approach will provide a more precise characterization of KYNA emission parameters as the disease progresses.

Another limitation of the study is the small sample size, particularly in the coenurosis group, which restricts the application of more advanced statistical techniques such as multiple analysis of variance or discriminant analysis. These methods would allow for a more comprehensive statistical assessment by considering the combined effects of multiple parameters, thereby enhancing the differentiation between infected and non-infected individuals. Future studies with larger sample sizes could greatly benefit from employing these more sophisticated statistical methods to improve the robustness of the findings.

## 5. Conclusions

This study provides evidence that KYNA levels in plasma may serve as diagnostic markers for chronic coenurosis in sheep. The reduction in KYNA levels in coenurosis-affected sheep could be linked to the neuroprotective role of KYNA and its involvement in neurological health. Early diagnosis facilitated by this tool could enhance management practices at the farm level by identifying infected animals before clinical symptoms become severe, allowing for timely interventions. Despite the lack of a direct treatment for sheep, the implementation of better diagnostic tools could significantly reduce the transmission of coenurosis and its economic impact on farms.

## Figures and Tables

**Table 1:** The results of the deconvolution process for the peak centered at ~388 nm associated with the emission of KYNA in the group of sheep without neurological symptoms (**A**) (n = 15) and with neurological symptoms (**B**) (n = 6).

AREA INT		FWHM		MAX HEIGHT		CENTER GRVTY		AREA INT P	
Group A	Group B	Group A	Group B	Group A	Group B	Group A	Group B	Group A	Group B
18,785	10,784	32	27	558	371	389	388	5.33	3.09
20,895	99,76	28.29	25	697	363	389	389	4.75	2.89
22,922	84,78	26.94	25	801	319	389	389	5.65	4.13
16,693	10,530	25.55	26	625	381	389	388	4.10	3.15
24,923	11,599	26	25	881	436	389	388	4.73	3.26
20,239	16,976	25	24	761	655	389	389	4.13	4.96
29,411		30		927		389		6.18	
22,469		26		814		389		4.98	
33,046		23		1312		389		4.65	
31,583		21		1380		388		3.72	
34,364		25		1301		389		4.37	
46,428		28		1565		389		6.23	
69,379		29		2260		388		7.91	
44,726		27		1565		389		7.21	
86,257		30		2671		388		10.15	

Area int: integrated area of the emission peak; FWHM: full width at half maximum; Max Height: maximum height of the emission peak; Center Grvty: center gravity; Area Int P: proportion of integrated area.

**Table 2:** Mean and standard deviation of blood plasma KYNA emission parameter for the group of sheep without neurological symptoms (**A**) and with neurological symptoms (**B**).

Parameter	Group A	Group B
AREA INT	28,225.27 ± 15,778.41	11,307.17 ± 3270.98 *
FWHM	26.85 ± 3.14	25.33 ± 1.37
MAX HEIGHT	1046.87 ± 501.31	419.17 ± 141.94 *
AREA INT P	5.12 ± 1.12	3.58 ± 1.11 *

Values are expressed as mean ± standard deviation, * *p* < 0.05. Area int: integrated area of the emission peak; FWHM: full width at half maximum; Max Height: maximum height of the emission peak; Area Int P: proportion of integrated area.

## Data Availability

The data present in this study are available within the article.
